# Measurement of the Total Lung Volume Using an Adjusted Single-Breath Helium Dilution Method in Patients With Obstructive Lung Disease

**DOI:** 10.3389/fmed.2021.737360

**Published:** 2021-09-08

**Authors:** Qing Liu, Lingxiao Zhou, Peiling Feng, Jinkai Liu, Bin Shen, Lili Huang, Yingying Wang, Yimin Zou, Yang Xia, Gang Huang

**Affiliations:** Key Laboratory of Respiratory Disease of Zhejiang Province, Department of Respiratory and Critical Care Medicine, Second Affiliated Hospital of Zhejiang University School of Medicine, Hangzhou, China

**Keywords:** obstructive lung disease, total lung capacity, the single-breath helium dilution, whole-body plethysmography, correction equation

## Abstract

**Background:** Whole-body plethysmography (WBP) is the gold standard for measuring lung volume, but its clinical application is limited as it requires expensive equipment and is not simple to use. Studies have shown that the single-breath helium dilution (SBHD) method, which is commonly used in clinical practice, significantly underestimates lung volume in patients with obstructive lung disease (OLD). By comparing the differences in lung volume measured using SBHD and WBP, we aimed to establish a correction equation for the SBHD method to determine the total lung volume in patients with OLD of different severities.

**Methods:** From 628 patients with OLD simultaneously subjected to SBHD and WBP, 407 patients enrolled between January 2018 and November 2019 were in the training group and 221 enrolled between December 2019 and December 2020 were in the prospective verification cohort. The multiple linear regression equation was used for data in the training group to establish a correction equation for SBHD to determine the total lung volume, and this was validated in the prospective validation cohort.

**Results:** There was a moderate positive correlation between total lung capacity (TLC) determined using the SBHD [TLC (SBHD)] and WBP methods [TLC (WBP)] (r = 0.701; *P* < 0.05), and the differences between TLC (SBHD) and TLC (WBP) (ΔTLC) were related to the severity of obstruction. As the severity of obstruction increased, the TLC was underestimated by the SBHD method. We established the following correction equation: *TLC* (*adjusted SBHD*) (*L*) = −0.669 + 0.756*TLC(*SBHD*)(*L*) – 0.047*$\frac{FEV1}{FVC}$+0.039*height (*cm*)–0.009*weight(*kg*)(r2 = 0.753 and adjusted r2 = 0.751). Next, we validated this equation in the validation cohort. With the correction equation, no statistical difference was observed between TLC (adjusted SBHD) and TLC (WBP) among the obstruction degree groups (*P* > 0.05).

**Conclusions:** The SBHD method is correlated with WBP to measure the total lung volume, but the SBHD method presents limitations in determining the total lung volume in patients with obstructive lung disease. Here, we established an effective and reliable correction equation in order to accurately assess the total lung volume of patients with OLD using the SBHD method.

## Introduction

The American Thoracic Society/European Respiratory Society (ATS/ERS) has proposed the following definitions of various lung disease patterns (1): (i) obstructive lung disease is defined as the forced expiratory volume in 1 s (FEV1)/vital capacity (VC) ratio below the fifth percentile of the predicted value; (ii) restrictive lung disease is described as a reduction in the total lung capacity (TLC) below the fifth percentile of the predicted value and a normal FEV1/VC ratio; and (iii) mixed lung disease was characterized by FEV1/VC and TLC below the fifth percentile of the predicted value. Generally, when the VC is reduced and FEV1/(F) VC is normal, it is used to infer the presence of restrictive lung disease; however, in ~40% of such cases, TLC is not reduced (2, 3). It is difficult to determine the exact nature and severity of a ventilation defect (obstructive, restrictive, or mixed) by measuring the FEV1 using only a spirometer without an accurate measurement of lung volume (1). Therefore, a precise lung volume measurement is essential to determine whether there are ventilation defects and provide good value for diagnosing and treating respiratory diseases (4, 5).

Various methods can be used to determine the TLC. The ATS/ERS guide proposes whole-body plethysmography (WBP), helium dilution, nitrogen wash-out, computed tomography (CT), and chest X-ray (CXR) to determine the TLC (4). WBP and gas dilution are the most commonly used methods to detect the lung volume in pulmonary function laboratories (6). The accuracy of the WBP method for measuring lung volume is not affected by a considerably uneven distribution of ventilation and is more accurate than other methods. However, to date, due to the high cost of the body box and complicated operation technology, its clinical application is limited. The single-breath helium dilution (SBHD) method is convenient, rapid, inexpensive, and widely used. However, this method assumes that the gas is evenly distributed in the lungs (4, 7–9), and the reliability of the test results is low in patients with a significantly uneven ventilation distribution. A regression equation of the SBHD method (10) was established to predict the multiple-breath TLC for patients with moderate to severe obstruction. Compared with SBHD, the test time of the multiple-breath helium dilution method is long, and the accuracy of measuring the TLC is high. But the two dilution methods underestimate TLC when compared with values determined by WBP which measures total thoracic volume regardless of the degree of ventilation of areas with severe air trapping. Owing to the aforementioned considerations, the purpose of our study was to compare the difference between the SBHD method and WBP in determining the total lung volume in patients with obstructive pulmonary disease and establish a correction equation to improve the accuracy of the SBHD method for measuring the lung volume in these patients.

## Materials and Methods

### Participants

Six hundred twenty-eight participants with obstructive pulmonary disease who undertook the SBHD and WBP tests were continuously enrolled from January 2018 to December 2020. We included 407 patients enrolled from January 2018 to November 2019 as the modeling group, and 221 patients enrolled from December 2019 to December 2020 served as the verification cohort.

The inclusion criteria were as follows: (1) age 18–80 years and (2) comply with the ATS/ERS definition of obstructive lung disease (2).

The exclusion criteria were as follows: (1) contraindications for spirometry, pulmonary diffusion function test, and WBP (1, 4, 5, 7) and (2) a history of lung surgery and recent history of chest trauma.

This study was approved by the institutional review board of Second Affiliated Hospital of Zhejiang University School of Medicine.

### Study Protocol

We recorded each participant's sex, age (years), weight (kg), and height (cm). All participants had an adequate pharmacological washout (short-acting bronchodilators were withdrawn 8 h, long-acting bronchodilators were withdrawn 48 h, and theophylline was withdrawn 24 h) before the start of the protocol. Each participant underwent spirometry and SBHD tests first, and then the WBP test after resting for 10 min (11). All lung function tests were conducted using a JAEGER spirometer (MasterScreen Body; Germany). We obtained predicted values of FEV1 for normal lung function and the lower limit of normal (LLN) of FEV1 from a nationwide study of reference values for spirometry in the Chinese population (12).

WBP: The participant sits in a body box, wears a nose clip, and breathes calmly. A valve is closed at the end of tidal expiration, and shallow and rapid breathing is required at a frequency of 0.5–1.0 Hz (13). Small changes in the lung volume and oral pressure were measured. As there was no gas flow in the airway, the oral pressure approximated alveolar pressure and the lung volume were calculated according to the Boyle's law.

SBHD: After the participant breathes calmly and steadily, they fully exhale to the residual volume position, evenly and quickly inhale the mixed gas to ≥90% VC, hold their breaths for 8–10 s, and then, exhale evenly and moderately to the residual volume position in 2–4 s. The percentage concentration of alveolar helium before and after dilution can be used to calculate alveolar volume, and the TLC can be obtained by adding the alveolar volume to the estimated dead space volume (4, 7).

### Result Processing

According to the ATS/ERS guidelines (1), the severity of obstruction is grouped according to FEV1%pred as follows: mild, FEV1%pred ≥ 70%; moderate, FEV1%pred 60–69%; moderately severe, FEV1%pred 50–59%; severe, FEV1%pred 35–49%; and very severe, FEV1%pred < 35%.

### Statistical Analysis

Statistical analysis was performed using SPSS Statistics 23.0 software (IBM SPSS, Armonk, NY, USA). A paired *t*-test was used to compare means between groups. The one-way analysis of variance was used to compare means among multiple groups, Wilcoxon test was used for non-parametric indicators, and Pearson correlation was used for correlation analysis. Results with *P* < 0.05 were considered statistically significant. Multiple linear regression analysis was used to establish a regression equation model for predicting TLC (WBP), and the variables included in the model were significant at *P* < 0.05. Bland-Altman plots were used to assess the agreement of the different methods for measuring the total lung volume.

## Results

### Overall Patient Characteristics and TLC Measurements

**Figure 1:**
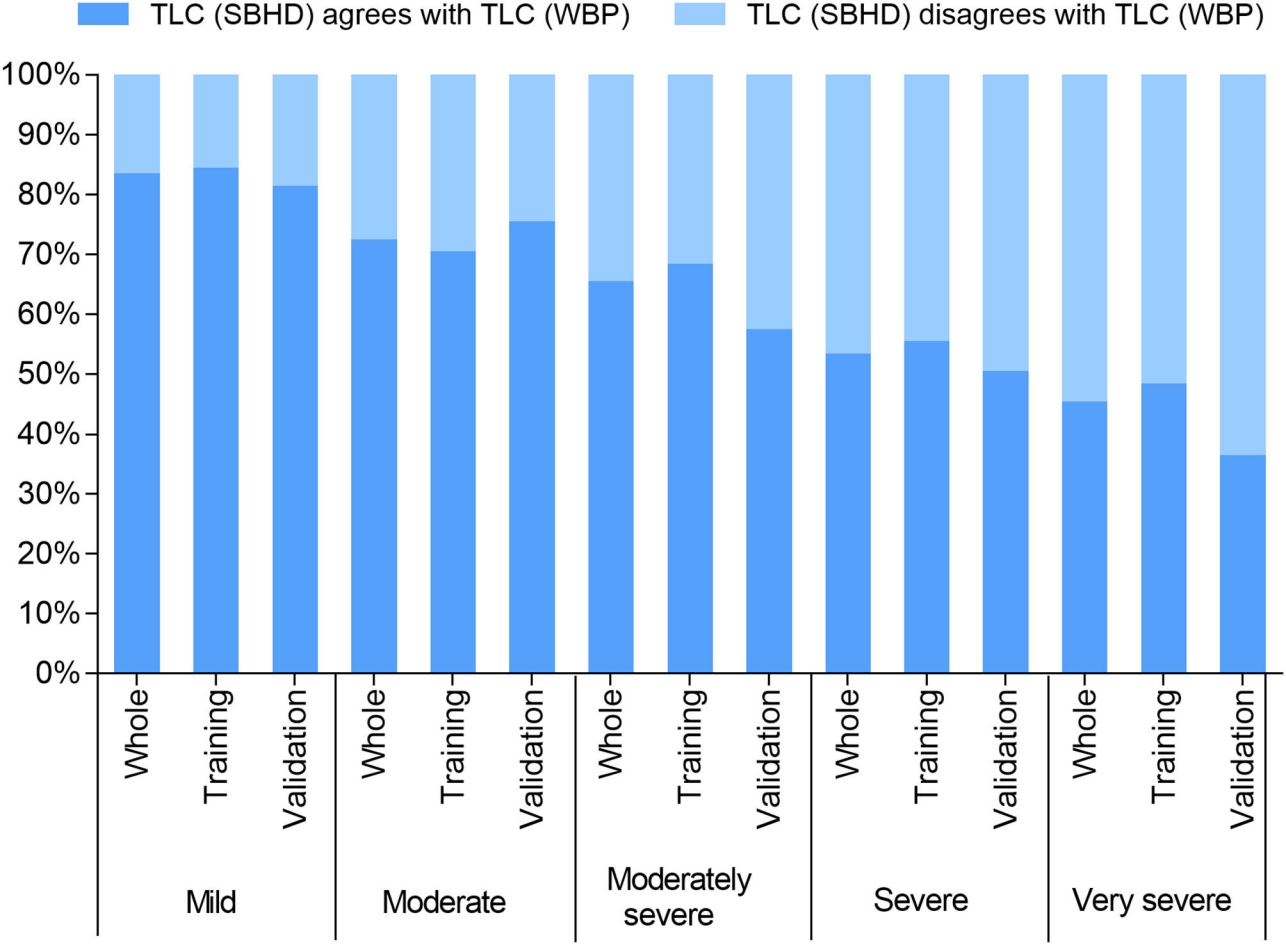
TLC (SBHD) agrees with TLC (WBP): represents equivalent indication of normal lung volumes between the methods (i.e., >LLN); TLC (SBHD) disagrees with TLC (WBP): represents the volume determined using the SBHD method was abnormally low (<LLN).

The baseline features and lung function results of the 628 subjects are shown in Table 1. The average age of the patients was 61.79 ± 11.1 years. The proportion of male patients was relatively high, ~3.5 times that of female patients. The ratio of patients in the five obstruction groups was similar(23.41%, 24.04%, 17.52%, 19.43%, and 15.60%). TLC (SBHD) (4.83 ± 0.94 L) was significantly lower than TLC (WBP) (6.30 ± 1.27 L; *P* = 0.000). There were no significant differences in sex, age, weight, or TLC between the training and validation groups. We further compared the results of the two methods to determine whether TLC was normal. We found that underestimation of TLC by the SBHD method easily caused misjudgment of lung disease patterns. We defined the misjudgment as: when the SBHD method judged TLC < LLN, but WBP judged TLC ≥ LLN, the proportion of this false result in the population. With the increase in the severity of obstruction, the misjudgment rate showed an upward trend, rising from 16 to 64% (Figure 1).

**Table 1:** Anthropometric characteristics and functional parameters in the 628 subjects.

**Variable**	**Whole**	**Training**	**Validation**
	**(*n* = 628)**	**(*n* = 407)**	**(*n* = 221)**
**Sex, %**			
Male	77.87	74.69	83.71
Female	22.13	25.31	16.29
Age, years	63.00 (56.00-70.00)	63.00 (54.00, 69.00)	64.00 (57.00, 71.00)
Height, cm	163.50 (159.00, 168.50)	163.00 (158.00, 168.50)	164.50 (161.00, 169.00)
Weight, kg	61.00 (54.00, 69.00)	60.00 (54.00, 68.00)	62.00 (55.00, 70.00)
BMI, kg/m^2^	22.94 (20.66, 25.01)	22.90 (20.55, 24.84)	22.99 (0.23)
**ATS/ERS classification**			
Mild	23.41%	20.39%	28.96%
Moderate	24.04%	22.11%	27.60%
Moderately severe	17.52%	18.43%	15.84%
Severe	19.43%	21.13%	16.29%
Very severe	15.60%	17.94%	11.31%
FVC, L	2.72 (2.28, 3.30)	2.70 (2.22, 3.31)	2.76 (2.40, 3.28)
FVC%pred	81.00 (68.93, 92.20)	80.56 ± 16.47	82.70 (71.05, 94.75)
FEV1, L	1.48 (1.04, 1.93)	1.40 (1.00, 1.83)	1.63 (1.17, 1.99)
FEV1%pred	58.8 (41.95, 68.50)	56.80 (39.20, 67.40)	61.37 ± 19.88
FEV1/FVC%	54.82 (44.18, 63.63)	53.53 (42.60, 62.15)	58.42 (47.37, 65.27)
TLC (SBHD)%pred	83.05 (75.30, 91.40)	82.90 (74.70, 91.20)	83.43 ± 12.43
TLC (WBP)%pred	108.25 (97.18, 118.43)	109.40 (98.70, 120.60)	105.50(96.40, 115.20)
ΔTLC, L	1.30 (0.79, 2.00)	1.35 (0.80, 2.08)	1.24 (0.77, 1.75)

*Data presented as median delta and 25-75% interquartile range or mean ± SD. BMI, body mass index; ATS/ERS, American Thoracic Society/European Respiratory Society; FVC, forced vital capacity; FEV1, forced expiratory volume in 1 s; TLC, total lung capacity; SBHD, single-breath helium dilution; WBP, whole-body plethysmography; ΔTLC: the difference between total lung capacity determined by whole-body plethysmography and total lung capacity determined by the single-breath helium dilution method*.

### Severity of Obstruction and TLC

**Figure 2:**
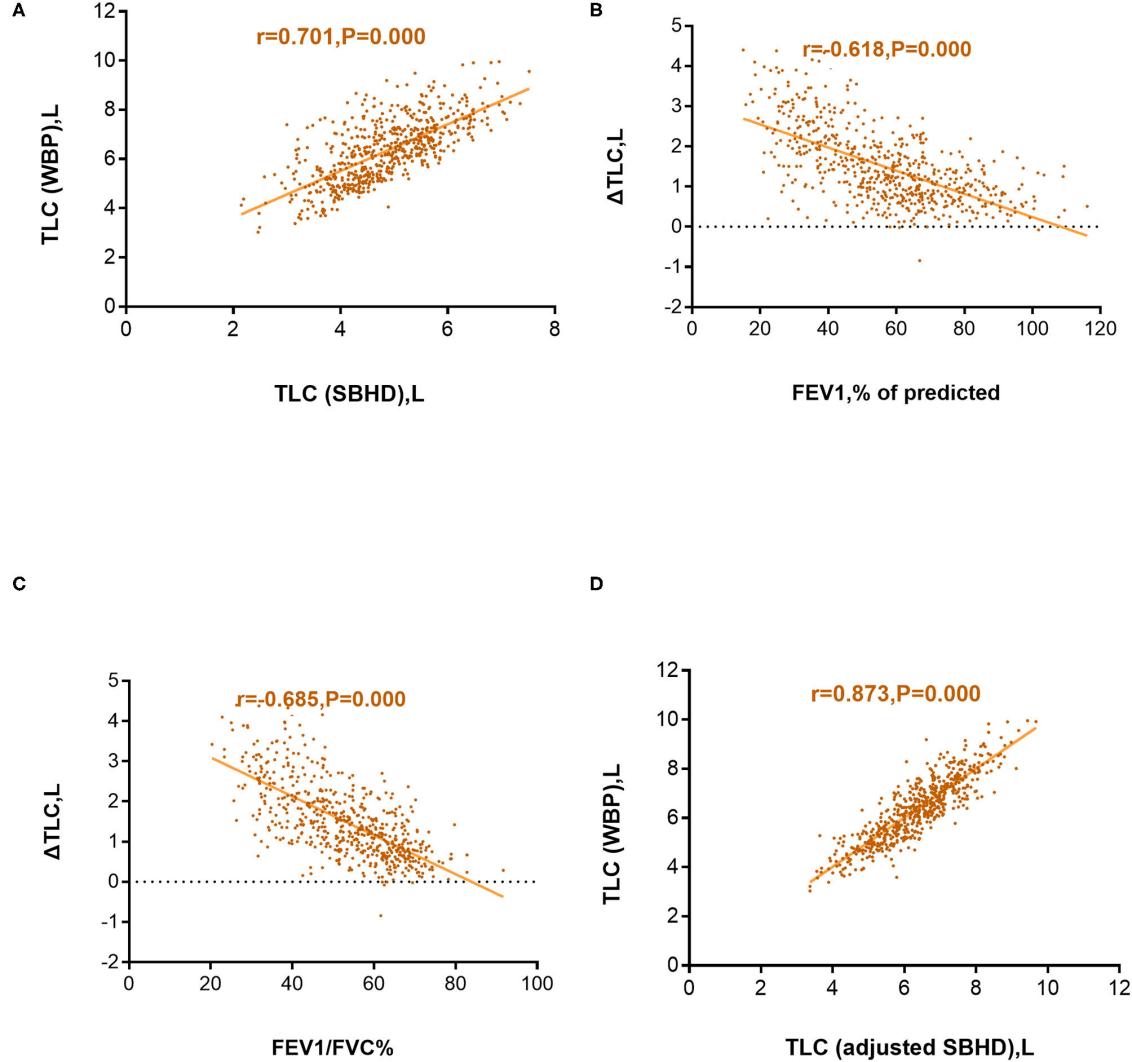
Correlation between the total lung capacity determined by whole-body plethysmography [TLC (WBP)] and total lung capacity determined using the single-breath helium dilution method [TLC (SBHD)] **(A)**; Correlation between the difference in TLC (WBP) and TLC (SBHD) (ΔTLC) and predicted forced expiratory volume in 1 s (FEV1%pred) **(B)**; Correlation between ΔTLC and FEV1/forced vital capacity (FVC%) **(C)**; Correlation between TLC (WBP) and TLC (adjusted SBHD) **(D)**.

We calculated the absolute difference between TLC (SBHD) and TLC (WBP) in each patient and defined it as ΔTLC. Pearson correlation analysis was used to evaluate TLC (SBHD) and TLC (WBP), as well as the correlations of ΔTLC with FEV1%pred and ΔTLC with FEV1/FVC. The results showed that TLC (SBHD) and TLC (WBP) were moderately positively correlated (r = 0.701; *P* = 0.000) (Figure 2A), the correlation coefficient between ΔTLC and FEV1%pred was r = −0.618 (*P* = 0.000) (Figure 2B), and the correlation coefficient between ΔTLC and FEV1/FVC was r = −0.685 (*P* = 0.000) (Figure 2C). After establishing the correction equation, the TLC (adjusted SBHD) and TLC (WBP) correlations were further improved (r = 0.873; *P* = 0.000) (Figure 2D).

### Establishment of the TLC (SBHD) Correction Equation

We compared TLC (SBHD) with TLC (WBP) in the training cohort of 407 cases, and the results showed that TLC (SBHD) (4.78 ± 0.95 L) was significantly lower than TLC (WBP) (6.31 ± 1.31 L; *P* = 0.000; Table 2), and multiple linear regression analysis was used to establish a regression equation for predicting TLC (WBP), including sex, age, height, weight, FEV1/FVC, and TLC (SBHD). In the multivariate analysis, sex and age were not significant and were excluded from the model. The resulting regression equation model is as follows: TLC (adjusted SBHD) (L) = −0.669 + 0.756 * TLC (SBHD) (L) – 0.047 * FEV1/FVC + 0.039 * height (cm) – 0.009 * weight (kg) (r^2^ = 0.753 and adjusted r^2^ = 0.751). Furthermore, after grouping by different degrees of obstruction, there was no statistical difference between the TLC (adjusted SBHD) and TLC (WBP) (Mild, *P* = 0.082; Moderate, *P* = 0.97; Moderately severe, *P* = 0.39; Severe, *P* = 0.53; Very severe, *P* = 0.99; Table 3).

**Table 2:** Differences (mean ± standard deviation) in TLC in different groups of subjects.

**Variable**	**Whole (*n* = 628)**	**Training (*n* = 407)**	**Validation (*n* = 221)**
TLC (SBHD), L	4.83 ± 0.94	4.78 ± 0.95	4.91 ± 0.90
TLC (WBP), L	6.30 ± 1.27	6.31 ± 1.31	6.27 ± 1.20
TLC (adjusted SBHD), L	6.29 ± 1.11	6.30 ± 1.14	6.28 ± 1.07
*P*-value	0.00	0.00	0.00
*P'*-value	0.82	0.98	0.88

*P-value indicates TLC (SBHD) vs. TLC (WBP); P'-value indicates TLC (adjusted SBHD) vs. TLC (WBP). TLC, total lung capacity; SBHD, single-breath helium dilution; WBP, whole-body plethysmography*.

**Table 3:** Differences (mean ± standard deviation) in TLC in the training group of subjects stratified by airflow limitation severity.

**Variable**	**Mild (*n* = 83)**	**Moderate (*n* = 90)**	**Moderately severe (*n* = 75)**	**Severe (*n* = 86)**	**Very severe (*n* = 73)**
TLC (SBHD), L	5.09 ± 0.85	4.78 ± 0.92	4.69 ± 1.08	4.73 ± 0.83	4.58 ± 1.03
TLC (WBP), L	5.99 ± 1.09	5.94 ± 1.12	5.93 ± 1.32	6.76 ± 1.25	7.01 ± 1.42
TLC (adjusted SBHD), L	5.91 ± 0.96	5.95 ± 1.03	5.98 ± 1.24	6.70 ± 0.91	7.01 ± 1.11
*P*-value	0.00	0.00	0.00	0.00	0.00
*P'*-value	0.082	0.97	0.39	0.53	0.99

*P-value indicates TLC (SBHD) vs. TLC (WBP); P'-value indicates TLC (adjusted SBHD) vs. TLC (WBP). TLC, total lung capacity; SBHD, single-breath helium dilution; WBP, whole-body plethysmography*.

### Validation of the TLC (SBHD) Correction Equation

We validated the aforementioned correction model in a validation cohort of 221 patients. We first compared TLC (SBHD) and TLC (WBP) in different obstruction degree groups. TLC (SBHD) was lower than TLC (WBP) (*P* = 0.000; Table 4), and this was consistent with the trend in the training group. We then applied the correction equation. The overall TLC in the validation group was 6.28 ± 1.07 L after correction, which was not statistically different from TLC (WBP) (6.27 ± 1.20 L; *P* = 0.88) (Table 2). Moreover, in the different obstruction degree groups, there were no statistically significant differences in TLC (SBHD) vs. TLC (WBP) after adjustment using the regression model (Mild, *P* = 0.58; Moderate, *P* = 0.82; Moderately severe, *P* = 0.10; Severe, *P* = 0.61; Very severe, *P* = 0.37; Table 4).

**Table 4:** Differences (mean ± standard deviation) in TLC in the validation group of subjects stratified by airflow limitation severity.

**Variable**	**Mild (*n* = 64)**	**Moderate (*n* = 61)**	**Moderately severe (*n* = 35)**	**Severe (*n* = 36)**	**Very severe (*n* = 25)**
TLC (SBHD), L	5.03 ± 1.05	4.96 ± 0.75	5.03 ± 0.83	4.68 ± 0.85	4.62 ± 0.97
TLC (WBP), L	5.85 ± 1.14	6.07 ± 1.13	6.34 ± 1.03	6.57 ± 1.20	7.29 ± 1.04
TLC (adjusted SBHD), L	5.82 ± 1.12	6.06 ± 0.88	6.49 ± 0.98	6.63 ± 0.93	7.17 ± 0.96
*P*-value	0.00	0.00	0.00	0.00	0.00
*P'*-value	0.58	0.82	0.10	0.61	0.37

*P-value indicates TLC (SBHD) vs. TLC (WBP); P' value indicates TLC (adjusted SBHD) vs. TLC (WBP). TLC, total lung capacity; SBHD, single-breath helium dilution; WBP, whole-body plethysmography*.

**Figure 3:**
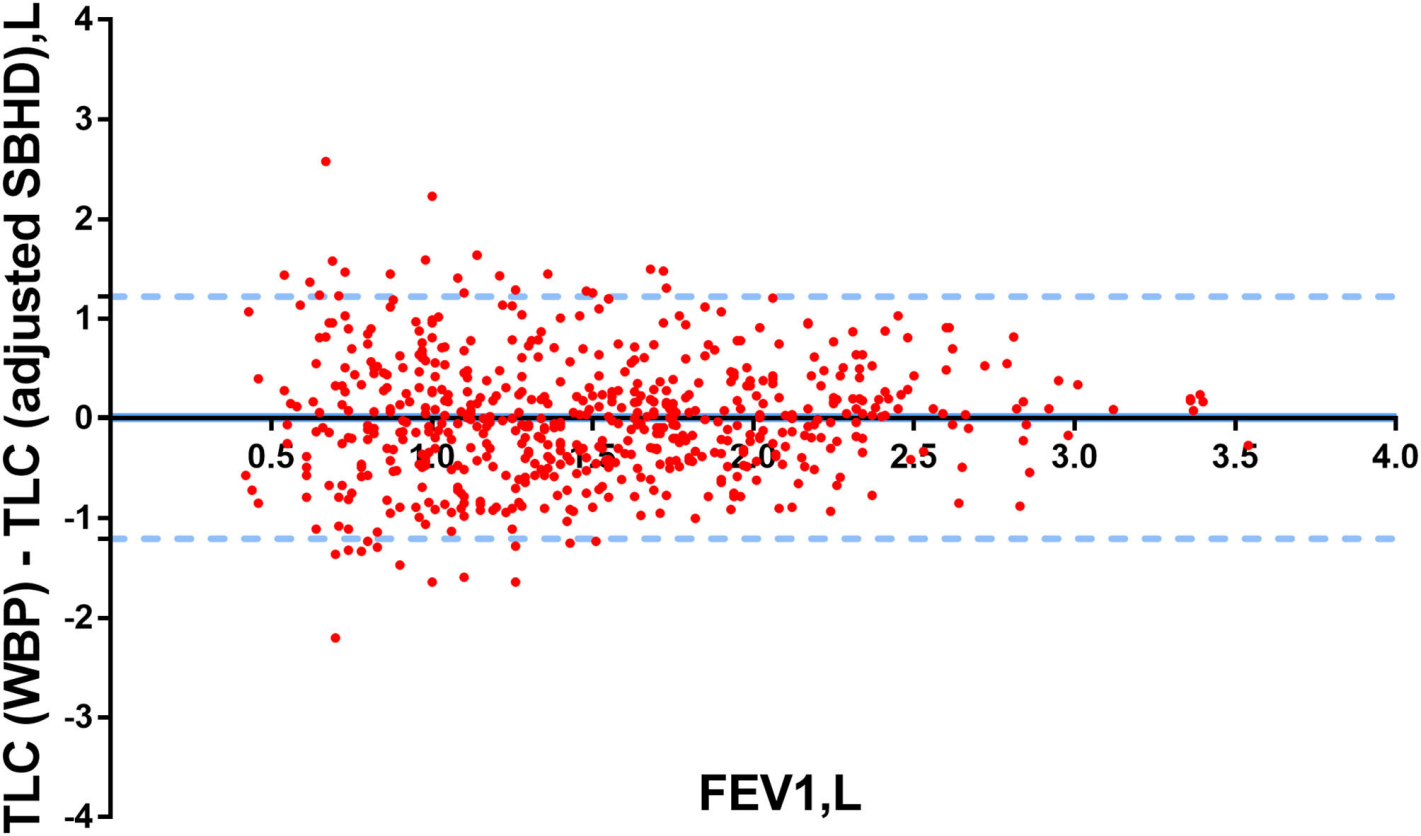
Bland–Altman plots were used to determine the differences in TLC (WBP) and TLC (adjusted SBHD) in patients. Y-axis: the differences between TLC (WBP) and TLC (adjusted SBHD), X-axis: FEV1 (L).The limits of agreement were calculated as ±1.96 SDs of the differences.

We used Bland–Altman plots to compare TLC (WBP) with TLC (adjusted SBHD) in all 628 patients. There was a very good agreement between TLC (WBP) and TLC (adjusted SBHD) in Figure 3.

## Discussion

In the study, we established a correction model for the SBHD method using WBP as the gold standard; to the best of our knowledge, our study sample size is currently the largest. We found that the SBHD method and WBP are moderately positively correlated in patients with obstructive lung disease, and the difference between the two gradually increases as the degree of airflow obstruction increases. We established a correction equation for the SBHD method, which allows SBHD to accurately assess the total lung volume in patients with obstructive pulmonary disease with different degrees of obstruction.

For patients with obstructive pulmonary disease, the SBHD method significantly underestimated lung volumes, which is consistent with previous findings. In a retrospective cross-sectional study, Coertjens et al. (14) found that the TLC (WBP) was higher than the TLC (SBHD) (*p* < 0.01). In groups of patients with mild to moderate OLD and severe OLD, the difference in TLC between the methods ranged from 1.58 to 2.00 L. Similarly, Milite et al. (15) measured TLC (SBHD), by the helium dilution rebreathing method [TLC (RBHD)] and TLC (WBP) in 55 outpatients with emphysema. In these patients with emphysema, TLC (SBHD) was underestimated compared with TLC (WBP) and TLC (RBHD), as FEV1%pred decreased (*P* < 0.001).

A possible explanation for the underestimation of the SBHD method is that the lung function of patients with OLD primarily manifests as airflow limitation. As the disease progresses, air trapping in the small peripheral airways, functional residual capacity or residual volume is significantly increased, airflow limitation causes compensatory responses in the body, the patient over-breathes, the chest cavity is overinflated, and the TLC is significantly increased (11, 16). The SBHD method calculates the total lung volume based on the percentage concentration of alveolar helium before and after dilution. Because patients with OLD have air trapping and uneven gas distribution, the inert gas cannot reach the area of air trapping. Thus, the TLC (SBHD) is lower than the actual value, because WBP can measure the trapped air not in communication with the airways in accordance with the Boyle's law. Therefore, WBP is usually preferred over the SBHD method in measuring the lung volume of patients with OLD.

An additional explanation of the discrepancy between WBP and SBHD methods is that plethysmography may overestimate TLC in airway obstruction due to a compliant extrathoracic airway (17). O'Donnel et al. (18) and Tantucci et al. (19) used an approach similar to ours and compared the TLC measured by WBP to that obtained by CT in patients. O'Donnell et al. (18) reported that WBP overestimates the lung volume, particularly among subjects with FEV1 < 30% of the predicted value. However, Tantucci et al. (19) arrived at an opposite conclusion. Their study showed that the results obtained by CT and WBP were similar, and the lung volume measured by WBP was not systematically overestimated. In our study, the data in Tables 3, 4 illustrate the rising TLC by WBP as the degree of airway obstruction gets worse. Although it is apparent that patients with OLD will have increased TLC, in our study, we instructed patients to take shallow and rapid breaths at a frequency of 0.5–1.0 Hz to reduce the oral pressure and alveolar pressure imbalance caused by wheezing frequency (13). Considering all studies and data currently available, WBP remains the most accurate method for determination of TLC.

The choice of methods for measuring the lung volume depends on cost, availability, convenience, and accuracy. Although the multiple-breath helium dilution method enables a more even distribution of the inhaled gas, the testing time is longer. In patients with severe airflow obstruction, the difference between the two helium dilution methods is as high as 34%, and the two still underestimated the lung volume compared with the total lung volume measured by WBP (20). CT can accurately calculate the anatomical lung volume (21, 22), but its usefulness may be limited by several factors, such as clinical conditions, radiation dose, and economic constraints (23, 24). A recent study of five global centers (25) proposed a new measure of absolute vital capacity, the Minibox™ method. The MiniBox™ is based on a combination of first principles and inductive statistics, by analysis of gas pressures and air flows immediately preceding and following airway occlusions, derives TLC during tidal breathing. The results showed no significant differences between the TLC values obtained by WBP and those using the Minibox™ method, but this new measurement method has not yet been included in the ATS/ERS guidelines and further research is needed to verify its accuracy.

The correction equation of TLC (SBHD) adjusted for the degree of airflow obstruction constitutes an important contribution of our study. Hopkins Asthma and Allergy Center (10) established a regression equation to show that the alveolar volume measured using the SBHD method can predict the multiple-breath TLC in patients with moderate to severe obstruction. In our study, a more comprehensive regression model was used, and the degree of difference between the WBP and SBHD methods was compared across severities of obstruction based on FEV1%pred, not across the FEV1/FVC ratio, as was done by Punjabi. In fact, assessing the degree of obstruction should not be based on FEV1/FVC, but instead on FEV1%pred; therefore, the current study is more accurate in this regard. Similarly, Coertjens et al. (14) established a correction for the SBHD method to measure the lung volume in patients with OLD; however, this study had a small number of patients, few included variables, a low regression model fit, and no validation cohort.

In our study, we included physiological factors, such as sex, age, height, and weight, in establishing the correction equation to improve the fit of the regression model. It is known that age, sex, weight, height, and ethnicity are the main physiological determinants of lung volumes (26–28). Height is positively correlated with lung volume (29), whereas obese subjects tend to show a decrease in lung volume with weight gain (30). This is consistent with our results. In our correction equation, TLC (adjusted SBHD) is positively correlated with height and negatively correlated with body weight. Although age and sex also had an effect on the lung volume (31, 32), the loss of significance of these two indexes in the process of establishing the correction model might be related to the low weighted value of sex and age indexes.

Our study had certain limitations. First, we only included test patients from a single hospital, and we will conduct multi-region and multi-center verification in the future. Moreover, the number of female patients was smaller than that of male patients, especially in the severe and highly severe groups. Domestic epidemiological surveys (33–35) have shown that the prevalence of chronic obstructive lung disease is significantly higher in men (11.9%) than in women (5.4%) and only 12.0% of people with COPD reported a previous pulmonary function test (33). This is mainly because the smoking rate in men (58.2%) is considerably higher than that in women (4.0%). In addition, women with COPD have a significantly lower inspection rate of pulmonary function test than men (35), and follow-up studies are needed to increase the number of female patients. Third, the population in our research involved all Chinese patients. We are not sure whether this correction equation is applicable to other ethnicities. The aforementioned questions will be addressed in future studies.

## Conclusions

In summary, in patients with lung disease affected by airway obstruction, the SBHD method underestimated lung volumes, and the difference between TLC (SBHD) and TLC (WBP) increased gradually with increasing degrees of airflow obstruction. After using the correction equation, the TLC (WBP) values can be more accurately predicted based on TLC (SBHD). The use of the correction equation makes the simple and low-cost SBHD method a reliable method for measuring the TLC of patients with OLD, providing important value in the diagnosis and treatment of respiratory diseases as well as in the course of observation and preoperative evaluation of patients.

## Data Availability Statement

The raw data supporting the conclusions of this article will be made available by the authors, without undue reservation.

## Ethics Statement

The studies involving human participants were reviewed and approved by the institutional review board of Second Affiliated Hospital of Zhejiang University School of Medicine. The patients/participants provided their written informed consent to participate in this study. Written informed consent was obtained from the individual(s) for the publication of any potentially identifiable images or data included in this article.

## Author Contributions

YX, GH, and YZ were involved in the conception and design. QL, LZ, PF, JL, BS, LH, YW, and GH were in charge of data acquisition, analysis, statistical analysis, and interpretation of data. QL, LZ, and YX were responsible for drafting and critical revision of the manuscript. All authors have read and approved the final manuscript.

## Funding

This work was supported by the Medical and Health Technology Program of Zhejiang Province (2018ZH013), Zhejiang Provincial Natural Science Foundation [LY20H010004], and National Natural Science Foundation of China [81870022].

## Conflict of Interest

The authors declare that the research was conducted in the absence of any commercial or financial relationships that could be construed as a potential conflict of interest.

## Publisher's Note

All claims expressed in this article are solely those of the authors and do not necessarily represent those of their affiliated organizations, or those of the publisher, the editors and the reviewers. Any product that may be evaluated in this article, or claim that may be made by its manufacturer, is not guaranteed or endorsed by the publisher.

## Abbreviations

ATS/ERS: American Thoracic Society/European Respiratory Society
CT: computed tomography
CXR: chest X-ray
FEV1: forced expiratory volume in 1 s
FVC: forced vital capacity
LLN: lower limits of normal
OLD: obstructive lung disease
SBHD: single-breath helium dilution
TLC: total lung capacity
TLC (WBP): total lung capacity determined by whole-body plethysmography
TLC (SBHD): total lung capacity determined by the single-breath helium dilution method
ΔTLC: the difference between total lung capacity determined by whole-body plethysmography and total lung capacity determined by the single-breath helium dilution method
WBP: whole-body plethysmography.
